# Mapping Evidence on the Effectiveness of Mind-Body Interventions and Brain Gym on Cognitive Function in Older Adults: A Scoping Review

**DOI:** 10.7759/cureus.104687

**Published:** 2026-03-04

**Authors:** Sandesh Sakpal, Suvarna Ganvir, Dipali Suvarna, Parag Ranade, Pothiraj Pitchai

**Affiliations:** 1 Department of Neurophysiotherapy, Dr. Vithalrao Vikhe Patil Foundation's College of Physiotherapy, Ahilyanagar, IND; 2 Department of Neurophysiotherapy, K J Somaiya College of Physiotherapy, Mumbai, IND; 3 Department of Neurophysiotherapy, Smt. Kashibai Navale College of Physiotherapy, Pune, IND; 4 Department of Community Physiotherapy, K J Somaiya College of Physiotherapy, Mumbai, IND

**Keywords:** brain gym, cognitive function, mind-body interventions, neuroplasticity, older adults

## Abstract

The increasing prevalence of dementia and age-related decline in cognitive function poses significant public health challenges. Brain Gym exercises and mind-body practices (MBPs), which are nonpharmacological interventions, enhance cognitive reserve and neuroplasticity through integrated breathing, meditative, and physical elements; however, in older adults with cognitive impairment, the evidence remains fragmented. Hence, this scoping review maps the evidence in older adults regarding the effectiveness of Brain Gym and MBPs for improving cognitive function, compares outcomes with conventional or no interventions, assesses feasibility and safety, and identifies research gaps while outlining recommendations.

A comprehensive search of PubMed and ScienceDirect (January 2020 to December 2025) identified English-language, full-text original research on MBPs versus comparators in community-dwelling or institutionalized adults. Five reviewers screened records, extracted data on study characteristics, interventions, and findings, and appraised quality using the Mixed Methods Appraisal Tool. A narrative synthesis approach was utilized to present the results. Eleven high-quality studies (n = 19-585; 2020-2025), primarily randomized controlled trials (RCTs) conducted in community settings across Asia, the US, Mexico, and Indonesia, were included. MBPs improved global cognition, memory quotients, executive function, and attention compared with usual care, with mixed superiority over aerobic comparators; Brain Gym enhanced brain-derived neurotrophic factor (BDNF) levels and domain-specific scores. Feasibility was high (81%-100% adherence, 89%-97% retention, no serious adverse events). Neuroimaging revealed gray matter increases in temporal and frontal regions and reduced inflammation. Gaps included short follow-up periods, limited virtual delivery, underrepresentation of frail subgroups, and limited mechanistic depth.

Thus, MBPs and Brain Gym demonstrate accessible and promising cognitive benefits via neuroplastic mechanisms, outperforming controls in feasibility and domain-specific gains. Multicenter, long-term studies with diverse, high-risk cohorts and hybrid modalities are essential to refine protocols, address equity, and support integration into geriatric care for dementia prevention.

## Introduction and background

Age-related cognitive decline is well documented in adulthood [1-4]. The prevalence of dementia is expected to rise from 46.8 million in 2015 to 131.5 million in 2050 globally [5]. Hence, a major public health challenge is the attenuation of age-related cognitive decline. Research has widely documented that nonpharmacological interventions focusing on physical activity are effective in improving cognitive function in older adults [6-8].

Mind-body practices (MBPs) are a broad term describing the close interaction between the body and the mind, encompassing practices that consist of meditative and physical components and potentially affect both behavior and brain function [9]. Unlike routine voluntary movements, which rely primarily on automatic neuromuscular pathways, MBPs intentionally integrate conscious mental processes, such as focused attention, mindfulness, or meditation, with controlled breathing and physical movements or relaxation techniques, thereby enhancing neuroplasticity and cognitive reserve beyond standard motor control [10]. There are several types of MBPs, such as yoga, tai chi, mindfulness meditation, Pilates, qigong, guided imagery, and massage therapy. Yoga is an ancient Indian mind-body practice that consists of physical movement (asanas), breathing exercises (pranayama), and meditation (dhyana) [11-13]. Tai chi is a Chinese martial art practiced primarily for defense training but also used for its meditative health benefits [14-16]. Pilates is another physical exercise method consisting of low-impact flexibility, muscular strength, and endurance movements [17-19]. Massage therapy uses hands-on manipulation of soft tissues to relieve muscle tension, reduce stress, and promote relaxation [20]. Qigong is a traditional Chinese MBP that combines gentle movements, controlled breathing, and meditation to cultivate vital energy (qi) and enhance well-being [21]. Guided imagery is a relaxation technique that employs mental visualization and positive suggestions to induce a focused, relaxed state and influence physiological responses [22]. These practices, by increasing muscular strength and body flexibility, help individuals gain various physiological and psychological benefits [23]. MBPs go beyond mere flexibility training. In addition to practicing the ability to move muscles and joints to their maximum range, they reduce tension in the body and mind, enabling practitioners to enter deeper meditative states [23].

Brain Gym is a collection of simple movements aimed at connecting or uniting the mind and body. It was developed by Paul and Gail Dennison in 1970 to improve various outcomes, including attention, memory, and academic skills [24]. Brain Gym is a kinesiology education program that has been promoted and applied in over 87 countries [24]. Kinesiology is defined as the study of body movement and the relationship between brain function and body posture. All the movements taught in Brain Gym are intended to enhance the learning process and integrate areas related to learning [25]. Brain Gym materials have been translated into over 40 languages [24]. Brain Gym interventions have been applied in children to improve academic performance [26,27]. However, it has also been suggested that Brain Gym may be considered a useful physical therapy strategy for older adults, as it may have a positive impact on brain function. A quasi-experimental study involving healthy older adults showed improvement in cognitive performance through specific patterns of movement and brain exercises similar to those described in the Brain Gym Manual [28]. A small randomized controlled trial demonstrated that Brain Gym intervention can enhance cognitive performance, specifically attention and memory, in older adults with dementia [29]. Similar results were reported by Mendrofan et al. [30] in a quasi-experimental study using a pre- and post-test design. In addition, a study involving 68 older adults who participated in Brain Gym for eight weeks reported improved sleep quality and reduced anxiety [31].

However, despite promising preliminary evidence suggesting that mind-body practices, including Brain Gym, may enhance cognitive function, there remains limited evidence to support claims regarding their benefits for older adults with cognitive decline. Hence, this scoping review synthesizes the existing literature on the effectiveness of mind-body interventions and Brain Gym exercises on cognitive function, mapping evidence, key concepts, and future research needs to advance this field.

## Review

The methodology for the present scoping review adhered to the Preferred Reporting Items for Systematic Reviews and Meta-Analyses extension for Scoping Reviews (PRISMA-ScR) [32] and the framework of Arksey and O’Malley (2005) [33]. The methodology involved five stages, which are outlined below [34].

Stage 1: identification of the research question

The purpose of this review was to map the evidence on the effectiveness of mind-body interventions and Brain Gym exercises for improving cognitive function in the elderly population. The primary research question was, "What is the current evidence on the effectiveness of mind-body interventions and Brain Gym on cognitive function in the elderly population?"

The secondary objectives were as follows: (1) What are the differences in cognitive outcomes between mind-body interventions and Brain Gym exercises compared with conventional or no interventions? (2) How do mind-body interventions and Brain Gym compare in terms of feasibility, adherence, and safety for cognitive enhancement in the elderly? (3) What are the gaps in research regarding intervention types, delivery modes (e.g., group vs. individual, in-person vs. virtual), and long-term effects on cognition? (4) What are the recommendations for future research and clinical practice?

Stage 2: identification of relevant studies

To capture contemporary evidence relevant to geriatric cognitive health practices, the search was limited to English-language, free full-text studies. PubMed and ScienceDirect databases were utilized from January 2020 to December 2025 to perform a comprehensive literature search. The search aimed to identify studies on the efficacy, safety, and outcomes of mind-body interventions for cognitive function in older adults. A combination of Medical Subject Headings (MeSH) terms and Boolean operators was used, consisting of ("mind-body interventions") OR ("mind-body therapies") OR ("brain gym") AND ("older adults") OR ("elderly") AND ("cognitive function").

Stage 3: selection of studies

The inclusion criteria were studies evaluating mind-body interventions and Brain Gym compared with conventional therapies, active controls, or no intervention; reporting outcomes related to cognitive function; including community-dwelling or institutionalized older adults; original research (e.g., RCTs, quasi-experimental, and cohort studies); and studies published in English with full-text availability. However, studies involving non-elderly populations, lacking full text or relevance, as well as review articles, opinion pieces, non-peer-reviewed articles, editorials, and conference abstracts, were excluded.

The population, concept, and context (PCC) framework focused on older adults in whom the effectiveness of mind-body interventions and Brain Gym on cognitive function was studied, along with outcomes, in clinical, laboratory, community, or home settings globally. Titles and abstracts were screened independently by five reviewers, and irrelevant studies and duplicates were removed. Full-text articles were then assessed for eligibility, with discrepancies resolved through discussion.

Stage 4: data charting

Study characteristics (authors, year, design, location, sample size, and population), intervention details (type, duration/frequency, delivery mode, and comparators), and findings on cognitive function, feasibility (e.g., adherence, adverse events), research gaps, and recommendations for future research were extracted. The Mixed Methods Appraisal Tool (MMAT) was utilised to assess the methodological quality of the included studies. Studies were classified as high, moderate, or low quality based on the tool’s specified criteria, which is a widely used instrument for assessing qualitative, quantitative descriptive (cross-sectional), nonrandomized, randomized controlled trials (RCTs), and mixed methods studies [35].

Stage 5: Collating, summarizing, and reporting the results

The results provided an overview of the effectiveness of mind-body interventions and Brain Gym on cognitive outcomes, highlighting differences from comparators, feasibility, implementation challenges, and proposed recommendations. A narrative synthesis was used to summarize findings via text, tables, and figures.

Results

**Figure 1:**
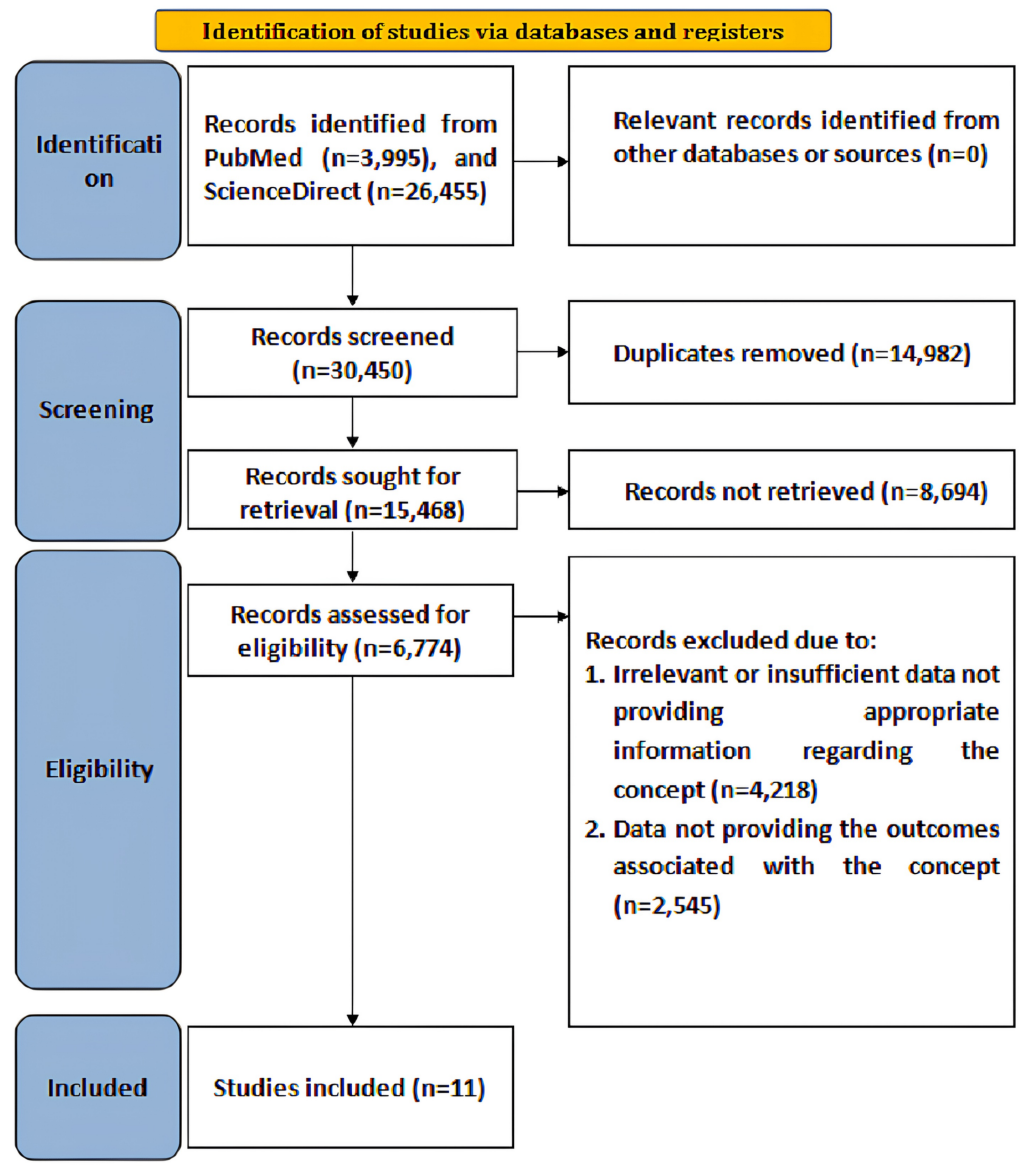
Search strategy

Initially, a total of 30,450 articles were identified after searching the databases from January 2020 to December 2025 using the specified keywords. Duplicate articles (n = 14,982) were removed after screening, leaving 15,468 articles sought for retrieval, of which 8,694 were not retrieved. Overall, 6,774 records were assessed for eligibility. Of these, 4,218 records were excluded due to irrelevant data that did not provide appropriate information regarding the concept, and 2,545 were excluded because they did not report the outcome measures specified for evaluation. Ultimately, eleven studies fulfilling the eligibility criteria were included in the current review [36-46]. The search strategy, according to the Preferred Reporting Items for Systematic Reviews and Meta-Analyses (PRISMA) flowchart, is illustrated in Figure 1.

The demographic characteristics of the included studies are reported in Table 1.

**Table 1:** Demographic characteristics of the included studies SD: standard deviation, RCT: randomized controlled trial.

Author and year	Study design	Study setting	Sample size	Population	Quality
Adriani et al. (2020) [36]	RCT	Indonesia (intervention site); Prodia Laboratories (laboratory examinations)	64 participants randomized (32 per arm); 58 completed (32 in Brain Gym® treatment group, 26 in control group).	Healthy Indonesian women aged >60 years.	High
Bhattacharyya et al. (2021) [37]	Longitudinal observational study	United States	2097	Adults (mean 64 years, standard deviation 11) at Wave 3, and (mean 55, SD 11) at Wave 2.	High
Yin et al. (2021) [38]	Pilot study	United States	Phase 1: Working group of 12 (seniors, community health workers, senior center staff). Phase 2: 56 participants enrolled (17 in the OfficialFAP (15 Latino participants), 19 in the ModifiedFAP (19 Latino participants), and 20 in the control group (15 Latino participants); 50 completed post-test (89.3% retention).	Community-dwelling low-income Latino adults aged ≥60 years (mean 74.9 years; SD 6.9).	High
Jiayuan et al. (2021) [39]	RCT	China	93 participants randomized (31 per arm); 91 (97.8%) completed follow-up (30 in mindfulness, which was Group 1, 31 in Tai Chi Chuan, which was Group 2, 30 in Mindfulness-Based Tai Chi Chuan, which was Group 3); 2 dropouts (1 refusal in Group 1, 1 hospitalization in Group 3).	Community-dwelling Chinese adults aged ≥65 years (mean 71.4 years; SD 4.6).	High
Zheng et al. (2021) [40]	RCT	China	69 participants randomized to magnetic resonance imaging substudy (23 per arm); 60 completed (20 per arm: 20 in Baduanjin group, 20 in brisk walking group, 20 in usual physical activity group); overall trial n=126 (42 per arm, per protocol paper).	Community-dwelling Chinese older adults aged ≥60 years (mean 65.5 years; SD 4.3).	High
Lenze et al. (2022) [41]	RCT	United States	585 participants randomized (146 to mindfulness-based stress reduction, 147 to exercise, 145 to combined MBSR + exercise, 147 to health education control).	Community-dwelling older adults aged 65–84 years	High
Cano-Estrada et al. (2022) [42]	Nonrandomized quasi-experimental study with pre- and post-test control group design	Mexico	30 participants (15 intervention, 15 control); all completed (no dropouts).	Institutionalized community-dwelling Mexican older adults aged ≥65 years (mean 70.73 years; SD 6.08).	High
Doorley et al. (2022) [43]	RCT	United States	19 participants (8 in Active Brains–Fitbit group, 11 in Health Enhancement Program group; 2 cohorts of 8–11 each); all completed (100% retention).	Community-dwelling older adults aged ≥60 years (mean 69.5 years; SD 6.2).	High
Chen et al. (2023) [44]	RCT	China	328 participants randomized (107 to Tai Chi Chuan group, 110 to fitness walking group, 111 to control group); 282 (86.0%) completed 36-week follow-up (analysis n=328).	Community-dwelling Chinese adults aged ≥60 years (mean 67.55 years; SD 5.58).	High
Xia et al. (2024) [45]	RCT	China	102 participants randomized (51 per arm); 91 completed (45 in the Baduanjin group, 46 in the usual physical activity control group; 11 dropouts).	Community-dwelling Chinese older adults aged ≥60 years (mean 70.3 years; SD 5.2).	High
Xu et al. (2025) [46]	RCT	Hong Kong	456 participants randomized (152 per arm); 423 included in analysis (139 in Combined mind–body physical exercise, cognitive training, and nurse-led risk factor modification group, 144 in nurse-led Risk Factor Modification group, 140 in health advice group).	Community-dwelling Chinese older adults aged 60–80 years	High

Moreover, the various findings on the effects of mind-body interventions and Brain Gym approaches on cognitive function, feasibility, and research gaps and future recommendations from the included studies are reported in Table 2.

**Table 2:** Summary of intervention characteristics, cognitive function outcomes, feasibility metrics, research gaps, and recommendations for future research from included studies on mind-body interventions and brain gym in older adults BDNF: brain-derived neurotrophic factor, BTACT: brief test of adult cognition by telephone, BMI: body mass index, NHIS: National Health Interview Survey, RCTs: randomized controlled trials, MBP: mind–body practices, SD: standard deviation, CHW: community health worker, mHealth: mobile health, MTCC: mindfulness-based Tai Chi Chuan, TCC: Tai Chi Chuan, CF: cognitive frailty, WMS-CR: Wechsler Memory Scale–Chinese Revised, BDJ: Baduanjin, UPA: usual physical activity, BWK: brisk walking, MRI: magnetic resonance imaging, MCI: mild cognitive impairment, COVID-19: coronavirus disease 2019, IQR: interquartile range.

Author and year	Intervention details	Findings on cognitive function	Feasibility	Research gaps	Recommendations for future research
Adriani et al. (2020) [36]	Brain Gym® (structured aerobic exercise involving head/eye movements and crossing extremities to stimulate both brain hemispheres, following Super Body, Super Brain protocol). Duration/Frequency: 12 weeks; twice weekly; 60 minutes/session (10-min warm-up, 40-min core movements, 10-min cool-down). Delivery Mode: In-person group sessions led by a professional instructor, ensuring correct movements. Comparators: (1) Brain Gym® (treatment); (2) Control (no intervention; participants did not join any course).	No significant between-group difference in primary outcome (Mini-Mental State Examination; 0–30, higher better scores at 12 weeks): Brain Gym® 25.93 ± 2.78 versus control 24.50 ± 3.36 (p=0.200). Baseline: no differences (25.03 ± 2.99 vs 23.42 ± 3.43, p=0.324). BDNF (brain-derived neurotrophic factor) increased in both groups but was significantly higher in Brain Gym® (41.26 ± 6.82 vs 37.10 ± 8.11 ng/mL, p=0.040; baseline: 39.43 ± 10.98 vs 33.61 ± 14.52 ng/mL, p=0.091).	High retention: 90.6% (58/64 completed; 6 control dropouts, reasons unspecified); no injuries or medical concerns reported during Brain Gym® sessions. Adherence: All treatment participants finished the program; no quantitative metrics (e.g., attendance rates) were reported.	Potential confounding from unrecorded additional physical exercise; unassessed genetic factors (e.g., BDNF gene polymorphism affecting synthesis); unclear if peripheral plasma BDNF reflects central levels in the elderly; limited to women (generalizability to men); short duration (12 weeks) may not capture long-term cognitive effects; no blinding (potential bias).	Investigate if peripheral plasma BDNF mirrors central levels in the elderly; evaluate Brain Gym® effects as a strategy for central degenerative disorders (e.g., via larger, blinded, longer-duration trials including men and diverse populations); explore dose-response, mechanisms (e.g., genetics, neuroplasticity), and integration with other interventions. Moreover, studies comparing the effectiveness of various mind-body interventions (e.g., yoga versus brain gym exercises) can be conducted.
Bhattacharyya et al. (2021) [37]	Observational exposure (not a controlled intervention): Movement-based mind-body practices (MBP; e.g., yoga involving physical movement/asanas, breathing/pranayama, meditation/dhyana, tai chi which is Chinese martial art for meditative health benefits, Pilates which is low-impact flexibility/muscular strength/endurance movements, assessed via self-report at Wave 2 ("In the past 12 months... how often did you use exercise or movement therapy which involve yoga, Pilates, tai chi, etc.?") on 5-point Likert scale: "a lot" to "never"; coded binary: any use (a lot/often/sometimes/rarely vs. none never; 16.8% any use). Type: Movement-based MBP (umbrella term for practices integrating physical movement, breathing, and meditative components). Duration/Frequency: Past 12 months (self-reported frequency not quantified beyond categorical; no dose-response data). Delivery Mode: Self-practice (not specified; habitual use for health/wellness). Comparators: No MBP (never used; 83.2%).	MBP use at Wave 2 independently associated with smaller decline in episodic memory (personal experiences tied to time/place; measured via BTACT immediate/delayed word recall) at Wave 3 (b=0.11, 95% confidence interval 0.01–0.20, p=0.03), after adjusting for socio-demographics (age, gender, race, marital status, education, employment), health/functional status (BMI); underweight/normal/overweight/obese, tobacco/alcohol use, chronic conditions count e.g., high blood pressure, diabetes, cancer, self-reported physical/mental health, activities of daily living (ADL)/instrumental activities of daily living (IADL) difficulties, depressive symptoms via DEPCON scale), conventional physical activity (moderate/vigorous), and baseline episodic memory (z-scored composite). No association with executive function (planning/organizing/reasoning/problem-solving; BTACT measures: inductive reasoning, verbal fluency, working memory span, processing speed, attention switching/inhibitory control; z-scored composite; b=0.03, 95% CI –0.03–0.08, p=0.34. Retest correlations: episodic memory r=0.53, executive function r=0.75 (higher stability). Frequency analyses: "Sometimes" use linked to better episodic memory change (b=0.17, p=0.03).	Not applicable (observational; no intervention delivered). Self-reported MBP use: 16.8% any in past 12 months (low prevalence, consistent with NHIS data: 14–15% for yoga/tai chi); no adherence/adverse event data.	Limited details on MBP duration/experience/dose-response (precludes causality/dose effects); broad MBP definition (e.g., yoga/tai chi/Pilates) may obscure type-specific impacts; recall bias in self-report (retrospective past 12 months); two time points limit non-linear trend analysis (e.g., accelerated decline); no initial cognitive impairment screening (potential undetected neurocognitive disorders); no medication data; focused on movement-based MBP only (excludes pure meditation like mindfulness); sample homogeneity (94% White; no racial/ethnic diversity analysis).	Conduct RCTs to test causality and dose-response of specific MBP types (e.g., yoga vs. tai chi) as non-pharmacological interventions for age-related cognitive decline; include more time points/shorter follow-ups for trend analysis; assess pure meditative practices (e.g., mindfulness, concentration-based meditation); replicate in diverse populations (e.g., racial/ethnic, international); explore neural mechanisms (e.g., hippocampal volume, prefrontal oxygenation, hypothalamus-pituitary-adrenal (HPA) axis, vagus nerve effects on inflammation/cerebral blood flow); integrate MBP with conventional activities for community-based cognitive health promotion.
Yin et al. (2021) [38]	Chinese Qigong mind-body exercise (adapted Five Animal Play/Wu Qin Xi: slow, imitative movements of tiger, deer, bear, ape, bird; includes breathing pranayama-like, meditation dhyana-like, and low-moderate exertion; Official traditional vs. Modified simplified for accessibility versions). Duration/Frequency: 16 weeks (biweekly 60-minute group sessions in weeks 1-4; weekly 30-minute phone calls in weeks 5-16); daily home practice encouraged (≥3 days/week, 20-30 minutes via video). Delivery Mode: Community health worker-led group sessions in-person at senior centers (initially; shifted to phone due to COVID-19); mobile health facilitated via Android tablet (26-minute video-guided practice), text reminders, bilingual handbook; culturally adapted (e.g., Spanish instructions, Latino-friendly themes). Comparators: Arm 1: Official Five Animal Play (traditional Qigong); Arm 2: Modified Five Animal Play (simplified Qigong; arms combined post-hoc); Arm 3: Placebo control (12-week healthy aging education program based on Aging Mastery, which involved topics like nutrition, advanced care planning).	Cognitive function assessed via Symbol-Digit Modalities Test (attention/speed) at baseline only (no posttest due to COVID-19 restrictions); no group differences or changes reported (mean baseline: 31.5; SD 10.6) overall; no significant intervention effects. Modest overall effects on secondary outcomes (e.g., quality of life via Short Form-36 mental component summary improved by 2.5 points (95% confidence interval 0.1-4.9); no specific cognitive domain improvements).	High feasibility: 89.3% retention with dropout reasons: COVID-19 (n=3), health (n=1); adherence: 79.4% met ≥70% weekly exercise goal (self-reported logs); session attendance: 80.1% for biweekly sessions, 61.3% for phone calls; fidelity: 100% CHW training completion, 95% protocol adherence; mHealth usability high (mean System Usability Scale 82.5/100); no adverse events reported; recruitment: 70% acceptance rate from screened (n=70 approached).	Limited evidence on Qigong efficacy in low-income Latino older adults; COVID-19 disruptions prevented full assessments (e.g., no post-test cognitive/physical function data, shortened in-person delivery to 4 weeks); small sample limits power for subgroup analyses (e.g., Official versus. Modified Qigong); no evaluation of cultural beliefs (e.g., Qi concept avoided to prevent confusion); placebo control may confound true effects; lack of long-term follow-up; understudied mHealth in older minorities with low digital literacy.	Conduct larger RCT comparing Five Animal Play Qigong to other low-impact exercises (e.g., Tai Chi, walking) without COVID-19 disruptions; include full pre/post assessments for cognitive/physical outcomes and long-term follow-up (≥6 months); evaluate scalability of CHW/mHealth delivery; assess cultural adaptations (e.g., Qi integration) and dose-response; target diverse subgroups (e.g., varying chronic conditions) for healthy aging promotion.
Jiayuan et al. (2021) [39]	Mindfulness-Based Tai Chi Chuan (integrates mindfulness, which involves present-focused, non-judgmental awareness via practices like body scan, walking meditation, with Tai Chi Chuan, which is an ancient Chinese exercise for body-mind harmony, using 24-simplified forms emphasizing slow movements, breathing, and balance. Duration/Frequency: 6 months total (3 months group: 2 sessions/week, 60 min each; 3 months individual: 2 sessions/week, 60 min each) + 6-month follow-up. Delivery Mode: In-person group sessions (10-min warm-up like stretching/joints, 45-min practice, 5-min cool-down such as breathing/relaxation; booklets provided); individual guided/supervised practice; themes progressed (e.g., mindfulness breathing to full MTCC sets); led by psychologists (mindfulness experts >10 years) and TCC specialists. Comparators: (1) Mindfulness alone (Group 1: formal/informal practices which involves body scan, walking meditation, gentle yoga, sitting meditation integrated into daily life e.g., mindful eating/hearing); (2) TCC alone (Group 2: 24-simplified forms e.g., "Cloud Hands," "Turn and Kick" with warm-up/cool-down); (3) MTCC (Group 3: modified TCC forms seated/standing/stepping with mindfulness focus on mind-body coordination).	Significant Group × Time interaction for primary outcome (cognitive frailty rate via Fried Frailty Criteria + CDR; χ²=6.37, p=0.041 at 12 months): MTCC reversal to no CF (non-frail (score 0) + CDR=0) 30% (9/30) vs mindfulness 6.7% (2/30), TCC 12.9% (4/31). Secondary: Mini-Mental State Examination (MMSE; 0–30, higher better) improved significantly (Group × Time F=8.456, p<0.05).	High retention (97.8%); adherence: 100% intervention completion; no adverse events; low dropout (2.15%); blinding maintained (independent teams for delivery/assessment); sample size achieved (powered for effect size 0.21, power 0.95).	No biochemical measures (e.g., biomarkers for CF mechanisms); limited to one province (generalizability); small evidence base for CF-specific interventions; no long-term (>12 months) outcomes; potential contamination risk despite separation.	Incorporate biochemical/subjective-objective indicators to explore mechanisms; expand multicenter trials across provinces/states for generalizability; larger samples to assess dose-response and long-term (>12 months) CF reversal; integrate MTCC into community health policies for scalable healthy aging.
Zheng et al. (2021) [40]	Baduanjin (traditional Chinese Qigong mind-body exercise: 8 symmetrical postures, e.g., "Two Hands Hold Up the Heavens," "Drawing the Bow to Shoot the Hawk" integrating slow movements, breathing techniques, and meditative focus for body-mind harmony). Duration/Frequency: 24 weeks; 3 sessions/week; 60 min/session (15-min warm-up involving stretching/joints, 40-min Baduanjin, 5-min cool-down which involves relaxation). Delivery Mode: In-person group sessions at community centers; led/supervised by professional coaches (per "Health Qigong Baduanjin Standard"); all groups received identical cognitive health education (30-min lectures/discussions every 8 weeks on nutrition/aging/cognitive decline); daily activity logs tracked usual physical activity. Comparators: (1) Baduanjin (mind-body exercise); (2) Brisk walking (aerobic: 55–75% heart rate reserve via Polar monitor); (3) Usual physical activity (control: maintain lifestyle).	No baseline group differences (except WMS-CR mental control subscore, p=0.019). At 24 weeks: BDJ vs UPA: significant improvements in MoCA total (24.30±1.78 vs 21.80±3.65, p=0.015), WMS-CR total (101.2±13.14 vs 87.30±20.69, p=0.029), memory quotient (WMS-MQ) (115.6±11.33 vs 103.2±19.78, p=0.037), mental control (9.60±2.35 vs 7.45±3.43, p=0.023), comprehension memory (9.90±1.68 vs 8.05±2.21, p=0.009) sub-scores. BDJ vs BWK: significant improvements in MoCA (24.30±1.78 vs 22.45±2.40, p=0.036), picture reproduction (10.50±2.14 vs 8.37±3.06, p=0.036) subscores. Gray matter volume (GMV) increases: BDJ vs UPA in left superior temporal/medial temporal gyrus, right superior/middle/medial frontal gyrus, right temporal medial/occipital medial gyrus, left cingulate gyrus, left superior parietal gyrus, right inferior parietal/angular gyrus (voxel p< 0.01, cluster p< 0.05); BDJ vs BWK in right frontal/precentral gyrus, right superior/medial occipital gyrus; BWK vs UPA in left thalamus, right frontal/corpus callosum/anterior cingulate gyrus, right occipital/precuneus, left inferior parietal gyrus. Right medial temporal gyrus GMV increase correlated with MoCA improvement (r=0.342, p=0.036, adjusted for age/gender/education).	Retention: 87% (60/69) for MRI substudy (9 dropouts: time conflicts); no adverse events related to interventions. Adherence: Moderate-intensity activity time significantly higher in BDJ/BWK vs UPA (1.45±0.71/0.97±0.75 vs 0.53±0.53 hours/day, p0.05).	Small MRI subsample (n=69); no participant blinding (expectancy bias possible); unquantified brisk walking intensity (may limit aerobic benefits); unknown MCI etiology (variable exercise response); limited generalizability (Chinese population only); no cellular-level mechanisms explored; short-term (24 weeks) follow-up; potential contamination from health education.	Larger, multicenter trials with quantified exercise intensity (e.g., peak oxygen consumption); longer follow-up (>24 weeks) for sustained effects; participant blinding strategies; explore MCI subtype-specific responses and cellular mechanisms (e.g., biomarkers); compare Baduanjin with other mind-body/aerobic exercises; diverse populations beyond Chinese older adults.
Lenze et al. (2022) [41]	Mindfulness-based stress reduction (MBSR; group-based program focusing on mindfulness meditation, body awareness, and stress reduction techniques). Duration/Frequency: 18 months total (6-month acute phase + 12-month maintenance phase). MBSR: 8 weekly 2.5-hour group sessions + 1 half-day retreat in acute phase, then monthly 2.5-hour sessions in maintenance; daily home practice targeting 60 minutes/day (guided audio). Exercise: Supervised 1.5-hour group classes twice weekly in acute phase, once weekly in maintenance; home practice targeting ≥300 minutes/week (aerobic, strength, functional movements). Combined: Both interventions concurrently. Delivery Mode: Primarily in-person (group classes at facilities); supplemented by home-based practice (e.g., audio recordings for MBSR, self-monitored logs for exercise). Comparators: (1) MBSR alone; (2) exercise alone; (3) combined MBSR + exercise; (4) health education control (matched group sessions on topics like nutrition/sleep, with discussion but no mindfulness/exercise goals).	No significant main effects or interactions on primary outcomes (composite scores for episodic memory and executive function SD 1; higher better) at 6 months: MBSR vs no MBSR (memory: mean difference –0.04 points (95% confidence interval–0.15 to 0.07), P=0.50; executive function: 0.08 points (–0.02 to 0.19), P=0.12); exercise vs no exercise (memory: 0.07 points (-0.04 to 0.17), P=0.23; executive function: 0.07 points (-0.03 to 0.18), P=0.17; interaction P ≥ 0.29). Similar null results at 18 months. Secondary outcomes (e.g., functional cognitive capacity via the Everyday Cognition scale, self-reported cognitive concerns) showed no significant improvements. Exploratory brain imaging (MRI): No benefits in hippocampal volume or dorsolateral prefrontal cortex surface area/thickness; unexpected greater hippocampal atrophy with mindfulness-based stress reduction (MBSR) at 18 months (mean difference -0.02 cm³ (95% CI -0.04 to -0.002), P=0.03).	High retention: 97.1% at 6 months, 81.2% at 18 months (lower in the control group, partly due to COVID-19 disruptions). Adherence: Median class attendance 90% ((IQR) 75%-100%) for MBSR and 83.3% (IQR 67%-92%) for exercise in the acute phase; home practice: 67% (IQR 45%-83%) of the target for mindfulness-based stress reduction (MBSR), 83% (IQR 57%-100%) for exercise. Per-protocol analyses (≥70% adherence) mirrored intention-to-treat results. No serious adverse events related to interventions; minor issues (e.g., muscle soreness) resolved without withdrawal.	Limited generalizability due to predominantly White (92%) and college-educated sample; focused on relatively healthy older adults with subjective concerns (not frailer or more impaired groups, e.g., with MCI or depression); potential need for longer durations to detect effects; unexplored mechanisms (e.g., cortisol, insulin sensitivity, aerobic fitness beyond self-report); unexpected hippocampal atrophy with mindfulness-based stress reduction (MBSR) requires replication; lack of diversity in socioeconomic/racial/ethnic representation; no assessment of long-term dementia prevention.	Evaluate interventions in more diverse (racial/ethnic, socioeconomic) or cognitively impaired populations (e.g., with MCI or comorbid depression/anxiety); test longer durations (>18 months) or tailored adaptations for subgroups; explore additional mechanisms (e.g., physiological markers like cortisol/insulin, advanced neuroimaging); conduct mediation analyses linking behavioral changes to brain outcomes; investigate combined or sequenced interventions for enhanced target engagement and cost-effectiveness in community settings.
Cano-Estrada et al. (2022) [42]	Brain gym (kinesiology-based simple movements to connect mind-body, improve attention/memory/academic skills; e.g., belly breathing, lazy eight, elephant, owl, arm activation, foot flex, cross-lateral walking). Duration/Frequency: 12 weeks; 2 sessions/week; 50 min/session (10-min warm-up (walking variations), 30-min general exercises (fine motor/balance/hand-eye coordination), 5-min cool-down (stretching)). Delivery Mode: In-person group sessions at gerontological centers, led by researchers (first session: cognitive function education); participants motivated via informed consent/explanation; control: matched educational talks on cognitive impairment. Comparators: (1) Brain gym intervention; (2) Control (educational talks only; retested 2 weeks post).	Significant within-group improvement in intervention group global Mini-Mental State Examination (MMSE; 0-30, higher better) score (baseline 22.7 ± 3.88 to post 25.3 ± 3.04, Z = -4.766, p < 0.001; normal range ≥24 achieved); domains: orientation (4.47 ± 1.06 to 4.87 ± 0.52 (temporal) and 3.73 ± 1.16 to 4.47 ± 0.74 (spatial), Z = -2.121/-2.37, p < 0.05), recall/memory (2.07 ± 0.96 to 2.53 ± 0.64, Z = -2.333, p < 0.05), language (7.87 ± 0.99 to 8.47 ± 0.74, Z = -2.310, p < 0.05); attention/counting improved (1.67 ± 1.59 to 2.00 ± 1.41) but non-significant (Z = -1.311, p > 0.05); registration unchanged (2.93 ± 0.26). No changes in control (global 21.2 ± 1.84 to 21.06 ± 1.67, p > 0.05; all domains p > 0.05). Multiple regression: education predicted 17.7% MMSE change variance (β = 2.899, p = 0.023; y = 15.740 + 2.899x); age was non-significant (r = 0.316, p = 0.047).	High feasibility: 100% session attendance/completion (no dropouts); all participants signed informed consent. No adverse events/injuries reported.	Small non-randomized sample (allocation by center chief; potential selection bias); confounding by education (intervention group more educated, possible residual effect on MMSE); no long-term follow-up (duration of effects unknown); limited generalizability (semi-rural low-resource Mexican institutionalized older adults); single cognitive test (MMSE influenced by age/education; cannot distinguish dementia types/mild cognitive impairment); few studies on brain gym in institutionalized/rural populations; scarce evidence on cognitive impairment in low-education/socioeconomic groups.	Larger randomized controlled trials with multiple cognitive tests (community-level, less age/education-biased); long-term follow-up (>12 weeks) for effect sustainability; explore in rural/semi-rural/low-resource populations; investigate confounding (e.g., education/social strata); develop/implement scalable non-pharmacological strategies (e.g., brain gym) for active aging/delaying decline; address demographic shifts via population-level interventions for quality of life.
Doorley et al. (2022) [43]	Active Brains-Fitbit (AB-F; group-based mind-body activity program emphasizing mindful movement (e.g., gentle yoga/tai chi-inspired poses), body awareness, breathing, and goal-setting for physical activity; integrated with Fitbit for step tracking/self-monitoring). Duration/Frequency: 8 weeks; 1.5-hour sessions/week (90 min live group + daily home practice). Delivery Mode: Fully remote/virtual via live video (Zoom; 6-8 participants/group led by trained facilitators (psychologists); Fitbit provided with app integration for activity tracking; educational materials via email/handouts). Comparators: (1) Active Brains-Fitbit (AB-F; mind-body activity intervention); (2) Health Enhancement Program (HEP; active educational control: matched time/dose with lectures on health topics (e.g., nutrition, sleep) + light stretching, no mind-body focus).	Preliminary signals of improvement in Active Brains-Fitbit (AB-F) group: Montreal Cognitive Assessment (MoCA; 0-30, higher better) increased by 1.5 points (95% confidence interval (CI) 0.2-2.8, p=0.03, within-group); no change in Health Enhancement Program (HEP) (0.1 points, p=0.89). Secondary: Improvements in perceived cognitive function (e.g., the Everyday Cognition scale decreased by 3.2 points (CI -5.8 to -0.6), indicating less impairment). No between-group comparisons due to small sample.	Met/exceeded a priori benchmarks (≥70% recruitment goal (100%), ≥80% retention (100%), ≥70% attendance (AB-F: 89% sessions, 78% daily practice; HEP: 92% sessions), ≥80% technology usability (95%); high satisfaction (Net Promoter Score 9.2/10)). No adverse events; minor technical issues resolved. Exit interviews: Themes of accessibility (e.g., "convenient from home"), engagement (e.g., "Fitbit motivating"), and preference for mind-body over education.	Small sample precludes between-group efficacy/power analyses; no long-term follow-up (>8 weeks); limited generalizability (predominantly White/highly educated; urban/coastal); unexamined mechanisms (e.g., neuroimaging, biomarkers); potential selection bias (self-selected motivated participants); no diverse chronic pain/cognitive decline subtypes; remote delivery may miss in-person social nuances.	Conduct fully powered remote efficacy RCT with larger/diverse sample (e.g., ≥100 participants, racial/ethnic minorities, rural/low-SES); include long-term follow-up (≥6 months), mechanisms (e.g., brain imaging, inflammation markers), and cost-effectiveness; compare to waitlist/usual care; adapt for subgroups (e.g., varying pain/cognition severity) and integrate with clinical care (e.g., primary care referrals).
Chen et al. (2023) [44]	Mind-Body Type: Tai Chi Chuan (TCC; 24-form simplified traditional Chinese mind-body exercise integrating mindful movements, breathing, meditation, and social elements for cognitive-physical harmony). Duration/Frequency: 24 weeks supervised + 12-week follow-up (total 36 weeks); 3 sessions/week; 60 min/session (10-min warm-up (stretching), 40-min practice, 10-min cool-down (relaxation)). Delivery Mode: Supervised in-person group sessions (10-15 participants/group) by certified instructors (≥5 years of experience, per China Tai Chi Chuan Association standards); moderate intensity (Borg scale 11-13); all groups received diabetes self-management education (30 min every 4 weeks for 24 weeks on nutrition, monitoring, and medication). Comparators: (1) Tai Chi Chuan (TCC; mind-body); (2) Fitness walking (FW; aerobic: continuous brisk walking at 60%-70% maximum heart rate via monitor); (3) Control (usual lifestyle + education).	Primary outcome (MoCA (Montreal Cognitive Assessment; 0-30, higher better) at 36 weeks, ITT): Tai Chi Chuan (TCC) superior to fitness walking (FW) (mean 24.67 (SD 2.84) vs 23.84 (SD 3.09); between-group difference 0.84 (95% confidence interval 0.02-1.66), p=0.046); both superior to control (22.95 (SD 3.32); differences 1.72 (95% CI 0.90-2.54), p control. Secondary: TCC improved memory quotient (MQ; Wechsler Memory Scale (WMS)) at 24/36 weeks vs control (differences 5.80 (95% CI 2.20-9.40), p=0.002; 4.26 (95% CI 0.55-7.96), p=0.024 vs FW at 36 weeks); attention (Digit Symbol Substitution Test (DSST)) and executive function (Trail Making Test Part B (TMT-B)) at 24/36 weeks vs control. Subgroups (36 weeks): Greater MoCA gains in women, body mass index (BMI) ≤24 kg/m², T2D duration >10 years, and ≤1 comorbidity.	High adherence: 88.8% (Tai Chi Chuan (TCC)) and 90.0% (fitness walking (FW)) attended ≥75% sessions (mean 64 (SD 5) sessions both, p=0.08); 92.4% completed 12 weeks, 88.1% at 24 weeks. Adverse events: 37 nonserious total (TCC: 8 (7.5%); FW: 13 (11.8%); control: 16 (14.4%); p=0.26); hypoglycemia: 0.9% (TCC), 1.8% (FW); falls: 4.7% (TCC), 9.1% (FW), 13.5% (control); no intervention-related serious events.	Community-dwelling only (excludes institutionalized/frail); potential expectancy bias (unblinded participants/instructors); short follow-up (36 weeks; no dementia progression data); resource-intensive (supervised sessions limit scalability); no neuroimaging/biomarkers (e.g., amyloid/tau); inconsistent prior exercise-cognition links in T2D/MCI; limited to Chinese population (cultural adaptation needed).	Larger, longer-term (>36 weeks) multicenter trials with diverse populations (e.g., non-Chinese, institutionalized) to assess dementia prevention; incorporate neuroimaging/biomarkers for mechanisms; test scalability (e.g., home-based, app-supported); blinded designs/active comparators; subgroup analyses (e.g., MCI subtypes, comorbidities); integrate with pharmacotherapy for comprehensive T2D/MCI management.
Xia et al. (2024) [45]	Mind-Body Type: Baduanjin (BDJ; traditional Chinese Qigong mind-body exercise: 8 symmetrical postures/movements (e.g., "Two Hands Hold Up the Heavens") integrating slow aerobic movements, breathing techniques, meditative focus for mind-body harmony, based on "Health Qigong Baduanjin Standard"). Duration/Frequency: 24 weeks; 3 sessions/week; 60 min/session (15-min warm-up (stretching/joints), 40-min Baduanjin, 5-min cool-down (relaxation/breathing)). Delivery Mode: In-person group sessions (15-20 participants/group) at local parks/community centers; supervised by 3 trained professional coaches; moderate intensity (58.86% maximum heart rate via Polar monitor); all groups received monthly 30-min health education (lectures on nutrition/aging). Comparators: (1) Baduanjin (BDJ; intervention); (2) Usual physical activity (UPA; control: maintain original lifestyle).	Primary outcomes (assessed at baseline, 13 weeks, 25 weeks): Significant Group × Time interaction for global cognitive function (MoCA (Montreal Cognitive Assessment; 0-30, higher better) score: increased by 2.51 ± 0.32 points in BDJ vs control, p< 0.001). Physical frailty (Edmonton Frail Scale (EFS; 0-17, higher worse) score: decreased by 1.94 ± 0.20 points in BDJ vs control, p=0.012; significant interaction p=0.012). Secondary outcomes: Improvements in body composition (e.g., reduced fat mass), grip strength, balance (e.g., Timed Up and Go (TUG) test), fatigue, specific cognition (memory via Wechsler Memory Scale-Chinese Revised (WMS-CR) subscores, executive function, visual-spatial), quality of life (Short Form-36 (SF-36)); structural/functional magnetic resonance imaging (MRI) showed increased gray matter volume/functional connectivity (e.g., hippocampal subregions). BDJ modulated oxidative stress (reduced malondialdehyde (MDA), increased superoxide dismutase (SOD)) and inflammation (reduced interleukin-6 (IL-6), tumor necrosis factor-alpha (TNF-α)).	High feasibility: 89.2% retention (11 dropouts: personal reasons/weather, no group difference p>0.05); adherence: 81.3% (average 57.7/72 sessions attended in BDJ; monitored via logs/heart rate); no significant adverse events reported; coaches not involved in recruitment/assessment to minimize bias.	Non-stratified randomization led to baseline imbalances (e.g., age); variable serum sample storage/timing may confound biomarker data (e.g., inflammatory cytokines); no active comparator (e.g., other exercises like jogging/swimming); small sample limits subgroup analyses; causality/mechanisms (e.g., oxidative stress/inflammation mediation) need confirmation; limited generalizability (Chinese community only); short-term (24 weeks) follow-up.	Use stratified randomization and timely biomarker testing in larger, multicenter RCTs with active comparators; explore long-term (>24 weeks) effects and mechanisms (e.g., mediation analyses for neuroimaging/oxidative stress); include diverse populations/international settings; assess cost-effectiveness/scalability for community healthy aging programs.
Xu et al. (2025) [46]	Mind-Body Type: Tai Chi (24-form simplified) as the mind-body physical exercise in the CPR (Combined mind-body physical exercise, cognitive training, and nurse-led risk factor modification) arm. Duration/Frequency: Overall trial: 15 months. Core interventions (CPR and RFM (Risk Factor Modification)): Quarterly nurse visits (30 min) + physician visits every 6 months (10 min). CPR-specific: Tai Chi (30 min/group, 3x/week for 12 weeks) + cognitive training (1 h/group, 3x/week for 12 weeks; activities: reading, mahjong, board/card games, discussions, painting, calligraphy, crafting, and music). Post-12 weeks: Self-practice encouraged via audio/video or groups. Delivery Mode: In-person at clinic (group-based for Tai Chi/cognitive training; individual for nurse/physician visits). RFM (Risk Factor Modification) tailored via motivational interviewing (Feedback, Responsibility, Advice, Menu Options, Empathy, and Self-Efficacy (FRAMES) guide) targeting vascular risks (e.g., diet: ≥2 fish portions/week, ≤50g sucrose/day; exercise; smoking cessation; QRISK3 target	No significant between-group differences in primary outcome (Alzheimer’s Disease Assessment Scale-Cognitive section (ADAS-Cog) Z-score at 15 months): CPR (Combined mind-body physical exercise, cognitive training, and nurse-led risk factor modification) vs. health advice (β = -0.04 (95% CI -0.34 to 0.26)); RFM (Risk Factor Modification) vs. health advice (β = -0.14 (-0.44 to 0.15)); CPR vs. RFM (β = 0.10 (-0.19 to 0.40); all corrected p > 0.05). Similar null results at 6/12 months. All groups showed within-group improvements in ADAS-Cog (Alzheimer’s Disease Assessment Scale-Cognitive section) and secondary outcomes (e.g., Clinical Dementia Rating Scale Sum of Boxes (CDR-SOB), Disability Assessment for Dementia (DAD)). Exploratory subgroups (e.g., baseline ADAS-Cog (Alzheimer’s Disease Assessment Scale-Cognitive section)/Physical Activity Scale for the Elderly (PASE)/15-item Geriatric Depression Scale (GDS-15)/dietary adherence medians; presence of hypertension/diabetes/hyperlipidaemia) showed no differential effects.	High nurse-led adherence: 94% (SD 13) in CPR (Combined mind-body physical exercise, cognitive training, and nurse-led risk factor modification), 96% (10) in RFM (Risk Factor Modification). CPR-specific: 59% (36) for Tai Chi sessions, 58% (37) for cognitive sessions; post-intervention continuation: 39% Tai Chi, 86% cognitive activities. One unrelated serious adverse event (distal sigmoid cancer in CPR). Retention: 93% completed ≥1 follow-up; dropouts were higher for partial completers (no significant baseline differences).	Limited RCTs on nurse-led RFM (Risk Factor Modification) alone for MCI cognitive outcomes; few studies integrate nurse roles in physical exercise/cognitive training; no prior multi-component interventions (e.g., CPR (Combined mind-body physical exercise, cognitive training, and nurse-led risk factor modification)) evaluated in primary care settings for MCI (Mild Cognitive Impairment); uncertainty on additive benefits of multi-component approaches over health advice in Asian primary care populations; short intervention duration (3 months for exercise/training) may limit detectability of effects.	Refine and re-evaluate multi-component interventions (e.g., CPR) in larger, longer-duration trials (>15 months) before recommending over standard health advice. Target high-risk subgroups (e.g., comorbid hypertension/depression) for tailored multi-domain approaches. Explore mechanisms (e.g., via compliance/PASE (Physical Activity Scale for the Elderly)/GDS (Geriatric Depression Scale)/dietary changes) and cost-effectiveness in diverse primary care contexts; prioritise Asian populations with MCI (Mild Cognitive Impairment) at elevated dementia risk.

Discussion

This scoping review synthesizes evidence from eleven high-quality studies exploring the effectiveness of mind-body interventions, including Brain Gym, yoga, tai chi, qigong variants, and mindfulness-based practices, on cognitive function among older adults. The included studies, conducted across diverse global populations in community and institutional settings, demonstrate promising yet heterogeneous outcomes. Several interventions often outperformed educational controls or usual care, enhancing memory, global cognition, attention, and executive function, while mixed superiority was observed when compared with aerobic exercises such as brisk walking. The studies demonstrated strong adherence and retention, reporting consistently high feasibility along with minimal adverse events. Modulation of biomarkers such as brain-derived neurotrophic factor and inflammatory cytokines, increased gray matter volume in frontal and temporal regions, and enhanced functional connectivity point toward neuroplasticity mechanisms. However, underrepresented subgroups, diverse delivery modes, and long-term effects remain gaps, highlighting the need for scalable and tailored approaches to address age-related cognitive decline.

A structured program of aerobic movements designed to stimulate brain hemispheres through head, eye, and cross-lateral exercises, known as Brain Gym, was evaluated by Adriani et al., who reported that peripheral levels of brain-derived neurotrophic factor, a key neurotrophin supporting neuronal survival, hippocampal neurogenesis, and synaptic plasticity, increased more substantially in the intervention group. This finding suggests that, through neurotrophic pathways, Brain Gym may foster cognitive reserve indirectly by potentially enhancing dendritic arborization and long-term potentiation without immediate macroscopic cognitive shifts. The retention and safety of the intervention affirm its potential as an adjunct for early cognitive maintenance; however, the absence of blinding and the focus on women limit mechanistic insights [36].

Bhattacharyya et al. investigated self-reported engagement in movement-based mind-body practices, such as yoga, tai chi, and Pilates, in a large observational cohort [37]. Regular practice was linked to attenuated declines in episodic memory, defined as the ability to encode and retrieve contextually rich personal experiences, but not executive functions such as reasoning or inhibitory control. Mechanistically, these practices may mitigate hippocampal atrophy through integrated effects on the hypothalamus-pituitary-adrenal axis, reducing chronic stress-induced glucocorticoid neurotoxicity, while promoting vagal tone to enhance cerebral blood flow and prefrontal-hippocampal connectivity [37]. The broad categorization of practices precludes identification of type-specific effects; nevertheless, the findings imply that habitual mind-body integration could buffer age-related memory decline via sustained anti-inflammatory and neurovascular adaptations [37].

Yin et al. adapted Chinese qigong's Five Animal Play, characterized by imitative, low-exertion movements blending breathwork and meditative focus, for low-income Latino older adults, comparing traditional and simplified versions with health education [38]. Although cognitive assessments were limited by external disruptions, secondary quality-of-life improvements suggested possible subtle attentional gains. Cultural tailoring of the intervention likely amplified engagement through resonant somatic awareness, potentially activating parasympathetic pathways to reduce sympathetic overactivity and preserve prefrontal resources for sustained attention. Self-efficacy was supported through mobile health facilitation via reminders and videos, suggesting that accessible, community health worker-led adaptations could leverage embodied mindfulness to address socioeconomic barriers to cognitive vitality. However, comprehensive mechanistic evaluation awaits larger trials [38].

By fusing nonjudgmental awareness practices with fluid, balance-oriented forms, rather than standalone mindfulness or tai chi, Jiayuan et al. evaluated mindfulness-based Tai Chi Chuan [39]. The combined approach demonstrated broader cognitive stabilization and excelled in reversing cognitive frailty. Mechanistically, this synergy may optimize dual-task neurocircuitry, enhancing default mode network deactivation during mindful movement to bolster error monitoring and working memory via anterior cingulate-insular integration, while tai chi’s proprioceptive demands may upregulate cerebellar-frontal loops for executive resilience. The intervention’s phased group-to-individual progression underscores its adaptability, positioning it as a holistic counter to frailty’s cascading neuromuscular-cognitive disruptions [39].

An eight-posture qigong sequence emphasizing symmetrical, breath-synchronized flows, known as Baduanjin, was assessed by Zheng et al. in individuals with mild cognitive impairment, relative to brisk walking or usual activity [40]. Baduanjin uniquely augmented memory quotients, global cognition, and subdomains such as comprehension and mental control, alongside gray matter expansion in the frontal, temporal, and parietal gyri. These volumetric changes likely originate from shear stress-induced endothelial nitric oxide release during rhythmic movements, leading to synaptogenesis and angiogenesis in memory hubs, complemented by meditative elements that downregulate amygdala hyperactivity to preserve attentional capacity. The coach-supervised structure of the protocol ensured fidelity, revealing Baduanjin’s edge over purely aerobic modalities in shaping brain architecture for integrated cognitive-physical harmony [40].

Lenze et al. reported that neither exercise nor mindfulness, nor their integration, yielded discernible gains in executive composites or episodic memory, with neuroimaging in the mindfulness arm revealing hippocampal thinning [41]. During heightened interoceptive training, this counterintuitive atrophy may reflect initial pruning of inefficient synapses, consistent with use-dependent remodeling. However, without parallel behavioral improvement, it raises questions about dosage thresholds for neuroprotection. The extended maintenance phase of the program and high fidelity highlight its tolerability, yet underscore the need to further examine stress-cognition pathways, such as cortisol-mediated hippocampal vulnerability, in less homogeneous cohorts [41].

Kinesiology-inspired Brain Gym movements were implemented in institutionalized older adults by Cano-Estrada et al. through figure-eight patterns, breathing, and coordinative drills targeting cross-hemispheric integration, in comparison with educational controls [42]. This led to enhancements in language domains, recall, and orientation, with education emerging as a modest predictor of responsiveness. While proprioceptive feedback loops may mitigate sensory deprivation associated with institutional settings, these simple gestures may recalibrate vestibulo-ocular reflexes to synchronize bilateral cortical activation, amplifying interhemispheric callosal transfer for spatial mapping and verbal fluency. Given the high adherence observed in a low-resource setting, Brain Gym may be considered a movement-mediated, equitable bridge to cognitive engagement in vulnerable older adults [42].

Doorley et al. piloted a virtual mind-body activity program incorporating gentle yoga-tai chi hybrids with wearable tracking for individuals with chronic pain and cognitive decline, compared with a health enhancement comparator [43]. Preliminary within-group findings suggested improvements in global cognition and perceived function. The remote format’s emphasis on embodied goal setting may harness operant conditioning via biofeedback, reinforcing mindful embodiment to interrupt pain-cognition interference cycles, possibly through descending inhibitory pathways that alleviate prefrontal overload. High usability and satisfaction affirm the role of digital mind-body interventions in fostering resilience, though mechanistic exploration of pain-modulated default mode network disruptions remains essential for scaling [43].

Chen et al. deployed simplified Tai Chi Chuan against fitness walking or usual care in older adults with type 2 diabetes and mild cognitive impairment [44]. Tai Chi outperformed walking in global cognition and memory, with amplified benefits in attention and executive tasks, especially among women and those with longer diabetes duration. Its meditative choreography may counteract the neuroinflammatory effects of glycemic fluctuations by elevating insulin-sensitizing adipokines alongside vagally mediated antioxidative cascades, thereby preserving thalamocortical relays for visuospatial executive demands. Supervised group dynamics further buffered isolation, positioning Tai Chi as a metabolic-cognitive stabilizer in comorbid aging [44].

Xia et al. applied Baduanjin to community-dwelling older adults with cognitive frailty, relative to usual activity [45]. The practice holistically improved global cognition, frailty indices, and domain-specific memory and executive functions, paralleled by gray matter accrual and biomarker shifts toward reduced oxidative stress and inflammation. These qigong flows likely orchestrate mitochondrial biogenesis in hippocampal neurons via Nrf2 pathway activation induced by coordinated breath and movement, curbing reactive oxygen species to safeguard synaptic integrity, while anti-cytokine effects dampen microglial priming associated with sustained neuroinflammation. The park-based accessibility of the protocol exemplifies community-embedded neuroprotection [45].

In comparison with risk modification or advice alone in mild cognitive impairment, Xu et al. integrated Tai Chi within a multifaceted regimen of MBE, cognitive games, and nurse-guided vascular risk modulation [46]. However, no arm-specific cognitive advantages emerged despite uniform within-group progress. The brevity of Tai Chi exposure may have constrained its kinesthetic imprint on prefrontal plasticity, potentially overshadowed by cognitive training-driven hippocampal recruitment or vascular risk interventions that enhanced endothelial function through lipid and glucose homeostasis. Nonetheless, the nurse-led hybrid model demonstrated strong uptake, indicating synergistic potential in primary care, where multimodal layering may cumulatively strengthen cerebrovascular reserve against progression [46].

Clinically, in geriatric protocols, these findings support integrating mind-body interventions as feasible and safe adjuncts to pharmacotherapy to promote holistic cognitive preservation, particularly for community-dwelling older adults with mild impairment or frailty. To democratize access across diverse settings, tailored delivery models, virtual for accessibility and group-based for socialization, can be implemented from urban clinics to rural centers, while nurse integration supports sustainability. In at-risk subgroups, future practice should prioritize early adoption to delay institutionalization and enhance quality of life, aligning public health priorities with personalized wellness.

Limitations

First, heterogeneity in populations, intervention modalities, and study designs complicates synthesis and direct comparison. Second, quantification of comparative efficacy across studies was not possible, as the synthesis did not include a meta-analytic evaluation due to the scoping review design. Third, generalizability to diverse high-risk groups is limited by modest sample sizes, which may have underpowered subgroup analyses. Fourth, follow-up durations were short, with limited assessment of long-term retention. Additionally, selection bias may have occurred due to the focus on free full-text articles and the English-language restriction from 2020 to 2025. Finally, few studies involved real-world validation, as most were conducted in clinic- or community-based settings.

Recommendations for future research

To assess sustained cognitive stabilization and domain-specific improvements, long-term follow-up is essential, incorporating prospective tracking of dementia progression alongside validated scales. Large-scale, multicenter RCTs evaluating integrated mind-body protocols, along with environmental adaptations such as virtual reality-enhanced sessions and functional cognitive tasks, should be conducted. Implementation studies should evaluate telerehabilitation adaptations to improve scalability, particularly low-cost, culturally tailored variants for underrepresented minorities. Moreover, comparative effectiveness studies between different types of mind-body interventions, such as yoga versus Brain Gym exercises, should be undertaken. To quantify mechanisms and equity impacts, systematic reviews with meta-analyses could be performed to guide policy for global cognitive health promotion.

## Conclusions

In older adults, this review highlights the transformative potential of mind-body interventions and Brain Gym as accessible and feasible strategies for enhancing cognitive function. These approaches surpass isolated activities by integrating kinesiology-based exercises, physical movement, breathwork, and meditative focus, thereby promoting neuroplasticity and cognitive reserve through elevated brain-derived neurotrophic factor levels, gray matter expansion, and reduced inflammation. Consistent gains in global memory, cognition, attention, executive function, and reversal of frailty were observed across studies. To curb cognitive decline and promote resilient longevity, further trials are needed to support the integration of mind-body approaches into public health frameworks as dementia prevalence continues to rise globally.
